# Prevalence of Intestinal Parasites and Associated Factors among Pulmonary Tuberculosis Suspected Patients Attending University of Gondar Hospital, Gondar, Northwest Ethiopia

**DOI:** 10.1155/2018/9372145

**Published:** 2018-02-15

**Authors:** Yalewayker Tegegne, Tadelo Wondmagegn, Ligabaw Worku, Ayalew Jejaw Zeleke

**Affiliations:** ^1^Department of Medical Parasitology, School of Biomedical and Laboratory Sciences, College of Medicine and Health Sciences, University of Gondar, Gondar, Ethiopia; ^2^Department of Immunology and Molecular Biology, School of Biomedical and Laboratory Sciences, College of Medicine and Health Sciences, University of Gondar, Gondar, Ethiopia

## Abstract

**Introduction:**

Intestinal parasitic infections are among the major public health problems in developing countries. Hence, it is significant to explore coinfection with intestinal parasites and pulmonary tuberculosis because coinfection increases the complexity of control and prevention of pulmonary tuberculosis and parasitic diseases.

**Objective:**

To assess the prevalence of intestinal parasites among pulmonary tuberculosis suspected patients.

**Method:**

Institutional based cross-sectional study was conducted at University of Gondar Hospital from March to May, 2017. Stool samples were taken from each participant and examined by direct microscopy and concentration technique. Descriptive statistics was performed and chi-square test was used to show the association between variables. *P* values of <0.05 were considered statistically significant.

**Results:**

Intestinal parasites were detected in 50 (19.6%) among a total of 256 pulmonary tuberculosis suspected patients who were included in the study, whereas the prevalence of pulmonary tuberculosis was 16.8% (43/256). Pulmonary tuberculosis and intestinal parasite coinfection was detected in 5 (2.0%) of the participants. The most prevalent intestinal parasites infection in this study was* Ascaris lumbricoides, *15 (5.85%), followed by* Entamoeba histolytica/dispar, *14 (5.46%), and Hookworm, 13 (5.1%).

**Conclusion:**

The prevalence of intestinal parasites and their coinfection rate with pulmonary tuberculosis among pulmonary tuberculosis suspected patients were considerable.

## 1. Background

Intestinal parasitic infections (IPIs) are among the major public health problems in developing countries and in many parts of the world. Globally, in 2010, an estimated 819.0 million, 438.9 million, and 467.6 million people were infected with* Ascaris lumbricoides*, hookworms, and* Trichuris trichiura*, respectively [1].

Epidemiological studies show that parasitic infections are among the most common infections and one of the biggest health problems worldwide. The prevalence of IPIs was ordinarily high in sub-Saharan Africa, and the incidence of IPIs is 50% in developed countries, but its incidence can range up to 95% in developing countries. These parasitic infections are caused by both protozoa and helminthes parasites. Diarrhoea is one of the most known clinical signs and symptoms of this parasitic infection [2].

In developing countries like Ethiopia due to inexperience of health promotion, low economic status, and poor level of environmental sanitation, IPIs remain the most difficult and unresolved public health problem. IPIs are commonly transmitted by unhygienic practice which includes ingestion of ova or cyst with unwashed hands, eating and drinking contaminated food, and reduced hygienic environments [3].

Based on recent World Health Organization (WHO) report, in 2015, globally around 10.4 million and 1.4 million people had developed PTB and died from all forms of TB, respectively. Almost one-fourth of the PTB cases were in the African Region. Ethiopia is placed eighth and second among twenty-two TB top burden nations of the World and Africa, respectively. The occurrence and death due to all forms of TB were 192 and 26/100,000-person population, respectively, in 2015. PTB continues to be one of the primary causes of morbidity and mortality because of transmissible diseases in Ethiopia [4].

Helminth infections induce Th2 immune responses which can involve different cytokines like IL-4, IL-5, and IL-13. This leads to an over time expansion of T cell regulation, which mainly have a negative impact on Th1 type of immune response to mycobacterium culture filtrate protein in humans and animal models with a return toward normal MTB-specific Th1 responses following complete treatment of the helminth infection [5, 6]. Moreover, a study conducted on animal models indicates that certain pathogens like parasitic coinfections are associated with more potent BTB (Bovine Tuberculosis) wounds in wild boar, because the availability of animals showing more potent BTB wounds has been associated with higher BTB prevalence in wild boar animals. According to this study a positive association was found between severity of BTB with coinfection of* Metastrongylus* species [7].

The increase in the prevalence of intestinal parasites among PTB patients indicated an increase in morbidity among these patients which implied the importance of continued stool examination and early treatment among PTB suspected patients [8].

Moreover, soil transmitted helminthiasis like* Ascaris lumbricoides* and hookworm can undergo heart to lung migration during their life cycle and this result in symptoms that may be confused with PTB [9, 10]. Thus, assessment of IPIs, mainly STHs (soil transmitted helminths), among PTB suspected patients is important for proper management of the patients. Besides, it is useful to assess the burden of coinfection between PTB and IPIs, since coinfection can increase the difficulty of prevention and control of PTB and intestinal parasitic diseases in coendemic regions [11, 12].

The prevalence of PTB and IPIs have been studied previously; however evidences are not enough concerning the extent of PTB and intestinal parasite coinfection in most of the provinces. Thus, the aim of this study was to determine the prevalence of smear positive PTB and IPIs and associated factors among tuberculosis suspected patients in order to provide valuable information of public health importance.

## 2. Methods

### 2.1. Study Design, Area, and Period

Institution based cross-sectional study was conducted at University of Gondar Hospital (UoGH) from March to May, 2017. UoGH is located in North Gondar Zone of Amhara Regional State, Ethiopia. Gondar is located 742 km far from Addis Ababa to Northwest Ethiopia. Its altitude is 2,200 m above sea level with an average temperature of 25 degrees centigrade. According to 2007 Ethiopian Census report, there are about 207,044 populations (98,120 male and 108,924 female) in Gondar town [13]. Currently the town has one referral hospital, UoGH, which is a teaching as well as referral hospital. It serves more than five million people in north Gondar zone and people of the neighbouring districts.

### 2.2. Study Population, Sample Size, and Sampling Procedure

Patients having cough of two weeks or more attending UoGH were source of population. Those individuals, who were selected using systematic random sampling technique were the study population/participants. However, individuals who have already started anti-TB treatment and participants who took antiparasitic drug during the two weeks before specimen collection were excluded. The minimum sample size was calculated using single population proportion formula (*Zα*/2)^2^(*p*)(1 − *p*)/*d*^2^, with the following assumptions: prevalence (*p*) of 26.3% from a previous study [14], 95% confidence level, and 5% margin of error. Accordingly, the minimum sample size (*n*) was found to be 293.

Study participants were selected based on the information we had on the number of PTB suspected patients attending UoGH in the study period. The monthly average number of clients attending TB clinic in the previous three months was 780. Then, the number of patients included in the study was distributed uniformly to each week (there were a total of 12 weeks and 60 working days in UoGH). Accordingly, the estimated number of PTB suspected patients attending UoGH TB clinic during the study period per week and per day was 65 and 13, respectively. Depending on this analysis approximate numbers of patients who were expected to attend TB clinic during the next three months (March to May 2017) of study period were determined. Finally, systematic random sampling technique (*k* = 780/256 = 3) was used to select study participants.

### 2.3. Data Collection and Processing

#### 2.3.1. Sociodemographic Data Collection

Sociodemographics like age, sex, and residence were collected by using a semistructured questionnaire by physicians through interview while the patient was attending tuberculosis outpatient department of UoGH.

### 2.4. Sample Collection and Laboratory Procedures

#### 2.4.1. Stool Specimen Collection and Processing for Parasite Examination

Patients were given a stool cup which was clean, dry, and leak proof labelled with a unique identification number and they were asked to bring about 5 g of stool sample. Then these stool samples were transported to UoGH Parasitology laboratory. Direct saline/iodine wet and formal-ether concentration technique were used for parasite investigation as described previously [15].

#### 2.4.2. Sputum Sample Collection and Processing

All study participants were given a screw-capped and labelled sputum specimen collection containers, and study participants were asked to bring three sputum samples, spot, morning, and spot sputum samples. Soon after sputum sample collection, smear microscopy was done by using florescence microscopy (FM) and Auramine O staining procedure which is described as follows: first a sputum smear was prepared and allowed to air-dry; then this smear was heat-fixed. After this the smear was stained using filtered 0.1% Auramine O solution for 20 minutes and washed properly and then decolorized with 0.5% acid-alcohol for 3 minutes and gently washed with flowing water. Finally it was counterstained with 0.5% potassium permanganate solution for 1 min and then properly washed with a flow of water and drained. At the end of the procedure, the back of the slide was cleaned and air-dried and the stained slides were examined under 20x and 40x magnifications of FM for acid fast bacilli (AFB) [15, 16].

### 2.5. Data Quality Control

The trustworthiness of the study results were assured by applying and following quality control measures during the total process of the laboratory procedures (preanalytical, analytical, and postanalytical quality control steps were followed). All materials, equipment, and procedures were adequately controlled. Negative and positive control slides were used to check the functionality of microscope as well as the accuracy of laboratory professional engaged in conducting the study. All slides were examined twice for confirmation of the result.

### 2.6. Data Management and Analysis

Data were collected and then entered and analyzed using Statistical Package for Social Sciences (SPSS version 20). Simple descriptive statistics was used to explain sociodemographic, prevalence rate of smear positive PTB, intestinal parasite, and their coinfection. Chi-squared test was used to compare the study variables for the presence or absence of association in all cases. *P* values of <0.05 were considered indicative of a statistically significant difference.

### 2.7. Ethics Approval and Consent to Participate

This study was carried out following ethical approval obtained from the research ethics review board of the University of Gondar, College of Medicine and Health Sciences. The permission was also obtained from UoGH supervisors. Informed verbal consent was obtained from each study participant. Any information obtained at each course of the study was kept confidential.

## 3. Results

### 3.1. Sociodemographic Characteristics of Study Participants

A total of 256 PTB suspected patients were included in this study with response rate of 89.8%. Out of 256 participants, 158 (61.7%) of the respondents were males and 98 (38.3%) were females. The study participants had a mean age range between 6 and 86 years with a mean age of 40.52 years. From all study participants, 176 (68.8%) live in rural area, whereas 80 (31.3%) live in urban (Table 1).

### 3.2. Prevalence of Intestinal Parasites and PTB among PTB Suspected Patients

Among 256 pulmonary tuberculosis suspected patients, intestinal parasites were detected in 19.6% (50/256).* Ascaris lumbricoides* and* Entamoeba histolytica* were the most frequently isolated parasites with the prevalence rates of 5.85% and 5.46%, respectively; whereas* Tania* species was the least, 1.17% (Table 2). Out of 256 PTB suspected study participants, 43 (16.8%) were smear positive for acid fast bacilli (AFB).

### 3.3. Coinfection of Intestinal Parasites and PTB among PTB Suspected Patients

The prevalence of coinfection of intestinal parasites and PTB was detected in 2% (5/256) of the study participants. Out of 5 PTB and IPIs coinfected patients, 0.8% (2/256) were PTB with hookworm and 0.8% (2/256) were with* Ascaris lumbricoides* (Table 3).

### 3.4. Factors Associated with Intestinal Parasites among PTB Suspected Patients

Age, sex, and residence site were considered for associated factor analysis. The prevalence of IPIs showed a significant difference among different age groups. The age group of 19–45 years was the most affected age group. For example, the prevalence of* Ascaris lumbricoides *and hookworm within this age category was 60% and 53.8%, respectively. Although there was no statistically significant association in the prevalence of IPIs with sex of the respondents, the prevalence of intestinal parasite was relatively higher in males 32 (12.5%) than females 18 (7.03%) (Table 4). Similarly, no statistical significant association was observed between IPI and residence. However, the prevalence of intestinal parasite in rural area was higher than that in those from urban area (12.1 versus 7.4%) (Table 4).

## 4. Discussion

The current study finding indicated that the overall prevalence of IPI among PTB suspected patients was found to be 19.6%. This finding is lower than a study conducted in different areas of Ethiopia, namely, Arba Minch (26.3%) [14], Northwest Ethiopia (29.0%) [17], and Gondar (28.9%) [18], and much lower than studies conducted in Brazil (57.8%) and Sub-Saharan Africa (40.3) [13, 14]. This observed difference might be due to difference in study period, method of stool examination, geographical area, and sample size. For example, recent deworming programs in school children for STH and schistosomiasis are being undertaken twice a year all over Ethiopia which might also minimize the new spread and burden of parasitic worms. On the other hand, a study conducted in China showed that it is relatively lower than the current study (14.9%) [19]. Differences on level of awareness about intestinal parasite transmission and prevention and economical status of the population might be essential determining factor for the decreased prevalence of intestinal parasite in China as compared to the current work.

*Ascaris lumbricoides* was the predominant parasitic infection in the study (5.9%). This finding was lower than that of a similar study reported from Arba Minch (11.3%) [14]. However, this finding is higher than that of a study conducted in China (0.5%) [19]. The prevalence of hookworm infection in the current study was 5.1%, which was lower than a study conducted in University of Gondar Hospital and poly clinic (11.1%) [18]. The difference in the prevalence of hookworm might be due to variations in the time of study, sample size, method of stool examination, and place of residence.

The present study suggested that there exists a significant detection rate of* Ascaris lumbricoides* and hookworm among PTB suspected patients. It is known that some developmental stages of these parasites involve heart to lung migration. Due to this fact, lung related symptoms (bronchospasm, fever, cough, dyspnoea, wheezing, and blood shade sputum) reported by patients might be due to these parasites [9, 10]. Thus, due consideration of such infections as a differential diagnosis is important.

In the current study,* Entamoeba histolytica* was the major intestinal parasitic protozoan infection (5.46%). This finding is slightly higher than a study carried out in China (1.4%) [19]. This might be due to difference in water supply, economic variation, feeding habit, environmental sanitation, and awareness of the ways of transmission and prevention and control measure of this parasitic infection.

In the current study prevalence of PTB and intestinal parasites coinfection was 2%, and the coinfection due to* Ascaris lumbricoides* and PTB was equivalent to that of the coinfection due to Hookworm and PTB. The finding of this result is in agreement with a study done in South India and Gondar [18, 20].

The overall prevalence of pulmonary tuberculosis cases in this study was 16.8%. The result of this finding is lower than similar studies carried out in Tanzania (81.2%) and South India (54%). The main reason for the differences in the prevalence of PTB in the current study and former studies might be due to the difference in diagnostic methods that were used for detecting AFB and sample size. For instance; in South India AFB were diagnosed and detected by Purified Protein Derivative (PPD) and culture, whereas in the diagnostic methods of AFB in Tanzania they were both microscopy and culture [20, 21].

The current study has also shown factors associated with IPIs among PTB suspected patients. The prevalence of IPIs showed a significant association among different age groups. The age group of 19–45 years was the most affected age group. This might be due to occupational related exposures of this age group. Even if there was no statistically significant association in the prevalence of IPIs with sex of the respondents, the prevalence of intestinal parasite was relatively higher in males than females. This result is in agreement with studies carried out in China, Arba Minch, and previous study conducted in Gondar [14, 18, 22]. In contrast to this finding, previous studies carried out in Kenya and China indicated that females were at higher risk of having IPIs than males [19, 23]. This variation of exposure among the different sex groups might be due to difference in occupational exposure in different communities.

### 4.1. Limitation

The major limitation of this study is that prevalence of IPI was determined by examination of single stool specimen from each study participant. Thus, we could not access the intra- and interstool variation of egg output. Furthermore, a single saline wet mount and formol ether concentration technique were examined for each of the stool specimens that may affect the accuracy of the egg count.

## 5. Conclusion

The current study has demonstrated that a significant number of intestinal parasitic cases were detected in varying degrees among TB suspected patients in the study area.* Ascaris lumbricoides* was the most common parasite isolated, followed by* Entamoeba histolytica* and Hookworm. Intestinal parasites and PTB coinfections were also identified and this may indicates that the patients' morbidity and mortality rates may be increased. Age was found to be an associated factor for IPIs among the study participants. Further case control and cohort studies need to be conducted to examine the association between intestinal* helminthes *infection and active tuberculosis.

## Figures and Tables

**Table 1:** Sociodemographic characteristics of PTB suspected patients at University of Gondar Hospital, Ethiopia, 2017.

Variables	Frequencies	Percentage (%)
Age		
5–18	19	7.4
19–45	140	54.7
46–65	73	28.5
>65	24	9.4
Sex		
Male	158	61.7
Female	98	38.3
Residence		
Urban	80	31.3
Rural	176	68.8
Total	256	100

**Table 2:** Prevalence of intestinal parasites among 256 PTB suspected patients at University of Gondar Hospital, Ethiopia, 2017.

Intestinal parasite	Frequency	Percent (%)
*A. lumbricoides*	15	5.9
Hook worm	13	5.1
*Tenia* species	3	1.2
*E. histolytica*/*dispar*	14	3.1
*S. mansoni*	5	2
Over all IP	50	19.6

**Table 3:** Prevalence of coinfection of intestinal parasites and PTB among PTB suspected patients at University of Gondar Hospital, Ethiopia, 2017.

Type of coinfection	Frequency	Percent (%)
Overall IPIs and PTB	5	2
*A. lumbricoides* & PTB	2	0.8
Hookworm & PTB	2	0.8
*S. mansoni* & PTB	1	0.4

**Table 4:** Factors associated with intestinal parasites among PTB suspected patients at University of Gondar Hospital, Ethiopia, 2017.

Variables	Intestinal parasitosis		Chi-square
	Yes, *n* (%)	No, *n* (%)	
Age			44.1^*∗*^
5–18	4 (3.5)	15 (98.4)	
19–45	30 (21.4)	110 (78.6)	
46–65	11 (15)	62 (85)	
>65	5 (20.8)	19 (79.2)	
Sex			0.137
Male	32 (20.2)	126 (79.8)	
Female	18 (18.4)	80 (81.6)	
Residence			1.32
Rural	31 (17.6)	145 (82.4)	
Urban	19 (23.8)	61 (76.2)	
PTB status			2.05
Positive for AFB	5 (11.6)	38 (88.4)	
Negative for AFB	45 (21.1)	168 (78.9)	

^*∗*^Statistically significant.

## Abbreviations

ATB: Active tuberculosis
BCG: Bacillus Chalmette Guerin
BTB: Bovine tuberculosis
UoGH: University of Gondar Hospital
IHI: Intestinal helminth infection
IPI: Intestinal parasite infection
LTB: Latent tuberculosis
MTB: * Mycobacterium tuberculosis*
OPD: Outpatient department
PTB: Pulmonary tuberculosis
STH: Soil transmitted helminths.
